# Prefrontal GABAergic Interneurons Gate Long-Range Afferents to Regulate Prefrontal Cortex-Associated Complex Behaviors

**DOI:** 10.3389/fncir.2021.716408

**Published:** 2021-07-12

**Authors:** Sha-Sha Yang, Nancy R. Mack, Yousheng Shu, Wen-Jun Gao

**Affiliations:** ^1^Department of Neurobiology and Anatomy, College of Medicine, Drexel University, Philadelphia, PA, United States; ^2^Institute for Translational Brain Research, Fudan University, Shanghai, China

**Keywords:** prefrontal cortex, interneurons, mediodorsal thalamus, ventral hippocampus, basolateral amygdala, complex behavior

## Abstract

Prefrontal cortical GABAergic interneurons (INs) and their innervations are essential for the execution of complex behaviors such as working memory, social behavior, and fear expression. These behavior regulations are highly dependent on primary long-range afferents originating from the subcortical structures such as mediodorsal thalamus (MD), ventral hippocampus (vHPC), and basolateral amygdala (BLA). In turn, the regulatory effects of these inputs are mediated by activation of parvalbumin-expressing (PV) and/or somatostatin expressing (SST) INs within the prefrontal cortex (PFC). Here we review how each of these long-range afferents from the MD, vHPC, or BLA recruits a subset of the prefrontal interneuron population to exert precise control of specific PFC-dependent behaviors. Specifically, we first summarize the anatomical connections of different long-range inputs formed on prefrontal GABAergic INs, focusing on PV versus SST cells. Next, we elaborate on the role of prefrontal PV- and SST- INs in regulating MD afferents-mediated cognitive behaviors. We also examine how prefrontal PV- and SST- INs gate vHPC afferents in spatial working memory and fear expression. Finally, we discuss the possibility that prefrontal PV-INs mediate fear conditioning, predominantly driven by the BLA-mPFC pathway. This review will provide a broad view of how multiple long-range inputs converge on prefrontal interneurons to regulate complex behaviors and novel future directions to understand how PFC controls different behaviors.

## Highlights

– PV-INs recruitment by MD inputs is crucial for working memory and social preference.
– SST-INs facilitate the coherence between vHPC-PFC during a working memory task.
– The vHPC inputs target on PV-INs in IL to inhibit CS-induced fear renewal.
– BLA innervates both PV- and SST- INs, but its function remains to be determined.

## Introduction

The prefrontal cortex (PFC) is well known for its top-down control of multiple distinct complex behaviors, including cognitive, emotional, and social behaviors, by selectively processing different input information (Carmichael and Price, 1996; Amodio and Frith, 2006; Hoover and Vertes, 2007; Yizhar and Levy, 2021). Multiple excitatory glutamatergic pathways are involved in controlling these complex behaviors with the PFC as an executive center. Canonically, the mediodorsal thalamus (MD)-PFC pathway is widely believed to be involved in controlling high-order cognitive performance, such as working memory, goal-directed behavior, and decision making (Mitchell and Chakraborty, 2013; Ferguson and Gao, 2015; Parnaudeau et al., 2018; Wolff and Vann, 2019). Whereas afferents originating from the ventral hippocampus (vHPC) are responsible for spatial working memory or navigation related tasks (Gordon, 2011; Neill et al., 2013). On the other hand, inputs from the basolateral amygdala (BLA) are thought to participate in expressing negative emotional behaviors such as fear, anxiety, and aggression (Nelson and Trainor, 2007; Arruda-Carvalho and Clem, 2015; Likhtik and Paz, 2015). However, the functions of these neural circuits are often not singular and complicated by overlapping roles in regulating specific behavioral components. This increases the complexity of categorizing the function of each particular pathway formed with the PFC.

As a convergent target of multiple long-range inputs, the medial PFC (mPFC) is required to precisely filter essential information from the numerous signals it receives from cortical and subcortical brain regions. Local GABAergic interneurons (INs) are critical for gating incoming long-range inputs. In the neocortex, interneurons comprise more than 20 molecularly-, morphologically- or physiologically-defined subpopulations, raising a major challenge in characterizing their regulatory function in controlling specific behaviors (Rudy et al., 2011; He et al., 2016; Paul et al., 2017). Among the cortical interneuron subpopulations, parvalbumin-expressing (PV-) and somatostatin-expressing (SST-) INs are the two most abundant subtypes. By taking advantage of newly developed transgenic mouse lines, researchers have comprehensively studied the distinct physiological properties of these two types of interneurons within the mPFC in recent years. Extensive research unveiled the unique abilities of PV- and SST- INs in gating inputs and controlling nearby pyramidal neurons (Cardin, 2018). PV-INs exert robust control over the information integration by targeting cell bodies and proximal dendrites of pyramidal neurons. In contrast, SST-INs enhance excitatory inputs’ selectivity by forming inhibitory synapses on distal dendritic branches of pyramidal neurons (Markram et al., 2004). Excitatory synaptic transmission onto PV- and SST- INs also exhibits differences in short-term plasticity (Gibson et al., 1999; Hofer et al., 2011). Briefly, the excitatory transmission is depressed on PV-INs but facilitated on SST-INs (Xiang et al., 2002; Ma et al., 2012). Therefore, different levels of the stimulation are required to activate PV- and SST- INs. A single burst of high-frequency stimulation is sufficient to excite PV-INs, resulting in brief but precise inhibition on targeted cell; whereas repeated stimulation is required to activate SST- INs, producing long-lasting and temporally delayed inhibition. Therefore, long-range inputs from a single brain region could play distinct and diverse roles in different complex behaviors by recruiting a separate subpopulation of INs. Indeed, due to their different excitabilities, PV- and SST- INs display distinct activity patterns in spatial working memory tasks (Kim et al., 2016). It is well established that all regions mentioned above, including MD, vHPC, and BLA, send projections to the PFC INs to form feedforward inhibition (Delevich et al., 2015; McGarry and Carter, 2016; Abbas et al., 2018). Here we review how each of those long-range afferents recruits particular INs to exert precise control of the mPFC local circuits involved in specific behaviors. We will dissect the anatomical connection of the MD, vHPC, and BLA with PV- or SST-INs in the PFC, demonstrate the roles of PV- and SST- INs in regulating these long-range inputs, and in mediating complex behaviors such as working memory, social interaction, and fear expression. Here, we focus on PV- and SST-INs as these two subpopulations are the largest expressed INs in the neocortex. The physiological and functional connection of long-range inputs to other types of interneurons, such as the vasoactive intestinal polypeptide expressing interneuron subtype, remains sparse and requires more studies to be characterized.

## Anatomical and Physiological Connections the Long-Range Afferents Formed With Prefrontal INs

Similar to other cortices, the mPFC also consists of laminar structures. Long-range inputs typically show laminar preference in the mPFC, with a higher proportion of them accumulating in layer I/II/III while layer V and VI mainly serve as output originators. Local interneurons also receive long-range inputs, which form the primary driving force of feedforward inhibition to the local circuits. Given the challenge in elucidating the multiple complex behaviors related to two different INs and three afferents, especially the technologies used in studying the enormous complexity of the relevant prefrontal cortical microcircuit, here we will focus on rodent studies. For the purpose of this review, we define the rodent mPFC as comprised of the anterior cingulate, prelimbic, and infralimbic cortex.

### MD-mPFC

**FIGURE 1:**
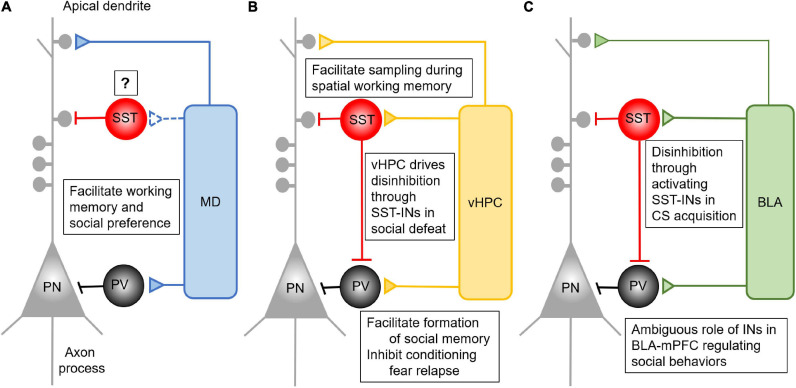
Anatomical and functional connectivity between mPFC and its major long-range inputs. **(A)** MD afferents form feedforward inhibition in the mPFC by driving the activity of PV- INs. This feedforward inhibition is critical for the performance of mPFC dependent working memory and social interaction, but its connection and role via SST-INs remains to be determined. **(B)** The vHPC afferents form feedforward inhibition in the mPFC by driving the activity of both PV- and SST- INs. Activation of prefrontal SST-INs by vHPC inputs facilitates spatial working memory performance. The inhibition of conditioning fear relapse is mediated by vHPC activating PV- INs, while vHPC activates SST-INs to inhibit PV-INs in the mPFC. This results in disinhibition of mPFC for the performance of social fear during the social defeat. **(C)** The BLA afferents form feedforward inhibition in the mPFC by driving the activity of both PV- and SST- INs. Presentation of CS in fear conditioning can activate SST-INs, which inhibit the PV-INs to form disinhibition in the mPFC. This process facilitates the acquisition of CS. Despite the importance of the BLA-mPFC pathway in regulating social behaviors, there is no concrete evidence elaborating the detailed function of PV- and SST-IN in it.

As corresponding thalamic and cortical partners, the MD and mPFC are reciprocally connected with one another (Heidbreder and Groenewegen, 2003; Mitchell and Chakraborty, 2013; Collins et al., 2018). Similar to the connection between sensory thalamic nuclei and their cortical partners, MD inputs can not only drive feedforward excitation but also inhibition on principal pyramidal neurons by activating PV-INs in the mPFC (Figure 1A). The activation of PV-INs by MD afferents is critical for maintaining the excitation/inhibition (E/I) balance in local prefrontal circuits (Anastasiades et al., 2018; Ferguson and Gao, 2018a). Using a retrograde tracing method and *ex vivo* electrophysiological recording, Delevich et al. (2015) found that the lateral and central part of the MD sends projections to layer 1 (L1), L3, and L5 of the dorsal anterior cingulate cortex (ACC), a subregion of the mPFC. These inputs directly target PV-INs to form a feedforward inhibitory circuit in the mPFC (Delevich et al., 2015), in support of a morphological study (Rotaru et al., 2005). Optical activation of MD afferents induces both monosynaptic excitatory and disynaptic inhibitory current in pyramidal neurons (Delevich et al., 2015). The monosynaptic excitatory postsynaptic currents (EPSCs) are detected on L3 PV-INs which are responsible for the disynaptic inhibitory current in pyramidal neurons. Interestingly, although both PV- and SST- INs fire action potential in response to activation of MD afferents in the mPFC, the latency of SST-INs is significantly longer than that of PV-INs. This finding suggested that SST-IN spiking might be driven mainly by local ploy-synaptic inputs rather than direct MD innervation (Delevich et al., 2015). However, a retrograde tracing study revealed that MD afferents form direct contact with SST-INs in the mPFC, although the literature has long been biased to PV-INs (Ährlund-Richter et al., 2019). The functional connection between MD afferents and prefrontal SST-INs needs further characterization in future studies.

### vHPC-mPFC

Although often overlooked in the literature, vHPC afferents form excitatory synapses not only on pyramidal neurons but also INs to promote feedforward inhibition (Figure 1B) (Thierry et al., 2000; Gabbott et al., 2002; Dégenètais et al., 2003; Dembrow et al., 2015; Liu and Carter, 2018; Marek et al., 2018). These inputs synapse on both PV- and SST- INs in the mPFC, and stimulation of vHPC inputs successfully induces EPSCs on both excitatory and inhibitory cells (Abbas et al., 2018; Phillips et al., 2019). In the mPFC, corticocortical projecting neurons in L5 preferentially receive inputs from the vHPC (Liu and Carter, 2018). Therefore, this subset of pyramidal neurons could be the primary regulatory target of the vHPC-driving feedforward inhibition in the mPFC. This assumption, however, remains to be tested. Further, optically activating vHPC inputs in the PFC triggers both AMPA and NMDA receptor-mediated EPSCs in fast-spiking PV-INs (Bogart and O’Donnell, 2018). Particularly, NMDARs in PV-INs play a critical role in forming functional connections between vHPC and mPFC during adolescent development (Alvarez et al., 2020). However, despite the well-founded anatomical connection of vHPC afferents to SST-INs (Sun et al., 2019), similar studies at the receptor level have not been done to characterize the synaptic properties in SST-INs in the mPFC. Activation of vHPC afferents will likely induce distinct responses in prefrontal SST-INs compared with PV-INs due to their different physiological properties and connectivity (Rudy et al., 2011; He et al., 2016; Paul et al., 2017), but this intriguing assumption remains to be determined.

### BLA-mPFC

The mPFC is a major top-down control center for the BLA in regulating the extinction of learned fear and other types of emotional behavior (Sotres-Bayon and Quirk, 2010). Anatomically, the mPFC and BLA are reciprocally connected (Gabbott et al., 2005; Hoover and Vertes, 2007). Although the BLA sends glutamatergic afferents to the mPFC, they preferentially target GABAergic INs to drive feedforward inhibition (Figure 1C) (Gabbott et al., 2005, 2006; Floresco and Tse, 2007; Dilgen et al., 2013; Klavir et al., 2017). Specifically, activating BLA inhibits most pyramidal neurons in the mPFC by activating PV-INs (Floresco and Tse, 2007; Dilgen et al., 2013), indicating a powerful inhibitory control of the prefrontal network activity by the BLA. McGarry and Carter (2016) reported that BLA inputs synapsed on cortico-amygdalar excitatory neurons but formed stronger connections with nearby PV- and SST- INs. Furthermore, these amygdalar-mPFC connections in PV-INs are more robust than those in SST-INs (McGarry and Carter, 2016). Interestingly, the presynaptic release of glutamate from BLA axon terminals is depressed on PV-INs but facilitated on SST-INs, enabling faster recruitment of PV-INs by BLA afferents (McGarry and Carter, 2016). Remarkably, both PV- and SST-INs fire action potential earlier than pyramidal neurons in the mPFC, suggesting that BLA inputs recruit INs faster to primarily trigger feedforward inhibition once activated (McGarry and Carter, 2016).

## Distinct Roles of Three Long-Range Afferents in the MPFC in Regulating Complex Behavior

### Working Memory

In rodents, working memory is a representation of an object, stimulus, or spatial location that is used to guide behaviors (Dudchenko, 2004). The working memory deficit is a primary cognitive symptom of schizophrenia (SZ) (Saykin et al., 1991, 1994; Park and Holzman, 1993) and other psychiatric disorders (Landrø et al., 2001; Martinussen et al., 2005; Arts et al., 2008; Lai et al., 2017). The dysfunction of mPFC, especially impaired GABAergic transmission, is believed to underlie the working memory deficits seen in SZ. Evidence from postmortem studies in SZ patients identified that both PV- and SST- INs displayed morphological (reduced immunopositivity), physiological (hypo-excitability), and molecular (reduced mRNA expression) dysfunction in the mPFC (Hartman et al., 2003; Hashimoto, 2008; Fung et al., 2010, 2014). It appears that SST- and PV- INs participate in different phases of working memory. Unlike PV-INs, which exhibit consistently increased activity during the delay period, SST-INs show more complex firing patterns with a strong target preference (Pinto and Dan, 2015). Direct inhibition of SST-INs, but not PV-INs, during the sample phase of the delayed non-match to sample T-maze task (DNMST) impaired performance (Abbas et al., 2018). Unlike the work done in 8-shape maze (Kim et al., 2016), inhibition of PV-INs in DNMST does not affect working memory performance (Abbas et al., 2018), which contradicts the canonical theory that prefrontal PV-INs play a more critical role in working memory. The discrepancy of functions between SST- and PV-INs during working memory are likely derived from different and specific upstream inputs. In this section, we depict how these long-range inputs regulate working memory through prefrontal INs.

#### PV-INs Regulate MD-PFC-Dependent Cognitive Function

The MD-mPFC pathway is widely involved in controlling high-order cognitive performance, such as working memory, goal-directed behavior, and decision making (Ferguson and Gao, 2015; Parnaudeau et al., 2018). Reduced functional connectivity between MD and mPFC is a central pathological mechanism underlying cognitive deficits in many neuropsychiatric disorders, including SZ (Block et al., 2006). A previous study discovered that the MD-mFPC pathway is critical for cognitive functional performance, decreasing MD activity impaired not only the flexibility in reversal learning, but also the ability to make correct choices in the DNMST (Parnaudeau et al., 2013). This study provides a piece of direct evidence for the role of MD-mPFC pathway in cognitive function. Recently, we reported that inhibiting MD activity by Designer Receptors Exclusively Activated by Designer Drugs (DREADDs, hM4Di) impaired the performance of the T-maze working memory task, which requires the information to be retained during varied delay periods (5, 15, or 60s) (Ferguson and Gao, 2018a). Activating PV- INs by a novel excitatory DREADDs via a parvalbumin promoter (PV-hM3Dq) successfully restored the working memory function impaired in long- (60 s) but not short- (5 and 15 s) delay trials. Presumably, activating PV-INs rescues working memory by correcting the disrupted E/I balance caused by compromised excitatory MD inputs to the mPFC (Ferguson and Gao, 2018a). Prefrontal pyramidal neuron activity features sequential firing during the delay period of working memory tasks (Bolkan et al., 2017; Schmitt et al., 2017). These pyramidal neurons’ sequential activity probably depends on both MD afferents and PV-INs driving feedforward inhibition. Although both PV- and SST-INs display high firing rates during the delay period of the working memory task (Kim et al., 2016), no study has been done to explore whether the activity of SST-INs in the mPFC is driven by MD inputs while performing the task. Interestingly, disrupting SST-INs activity could affect mouse working memory performance in relatively short-delay trials (10 s) (Kim et al., 2016). Collectively, this evidence suggests that SST-INs may mediate short delay working memory, but the role of MD inputs remains to be determined.

#### SST-INs Facilitate vHPC-mPFC Synchrony and Prefrontal Spatial Coding

Besides the MD-mPFC pathway, synchronization between mPFC and vHPC is also important for the performance of working memory. The communication between vHPC and mPFC through synchronized oscillations critically regulates spatial working memory (Gordon, 2011), whereas functional dissociation between these two brain regions is an important feature of SZ etiology (Ford et al., 2002; Meyer-Lindenberg et al., 2005; Esmaeili and Grace, 2013; Alvarez et al., 2020). Spellman et al. (2015) demonstrated that optically inhibiting the vHPC projection to the PFC impaired spatial working memory performance in mice. Interestingly, the power of both theta and gamma-band oscillations are increased in both mPFC and HPC in spatial working memory tasks (Jones and Wilson, 2005; Sigurdsson et al., 2010; Neill et al., 2013; Hallock et al., 2016; Lagler et al., 2016). INs are crucial for oscillation synchrony between these two long-range connected regions. Indeed, Abbas et al. (2018) found that inhibiting SST-INs during the sample phase, when the cue was presented to animals, impaired spatial working memory performance. More interestingly, the phase-locking between mPFC single-unit activity and theta oscillation in the vHPC is decreased when SST-INs were inhibited during the sample phase. It is thus possible that GABAergic INs facilitate the synchronously enhanced theta and gamma-band oscillations between mPFC and vHPC during spatial working memory. By doing this, SST-INs would be expected to encode information during the sample phase through facilitating the communication between vHPC and mPFC, which is critically important for the subsequent delay period neural activity. Inhibition of SST-INs activity during the sample phase leads to disinhibition of pyramidal neurons, which could continuously fire action potentials in the subsequent delay phase of the spatial working memory task. Altogether, there is strong evidence supporting the conclusion that SST-INs gate the vHPC inputs to the mPFC to encode spatial information in the spatial working memory task (Abbas et al., 2018). In contrast, inhibiting PV-INs at any phases did not affect phase-locking activity to the vHPC theta nor working memory accuracy (Abbas et al., 2018). One possible explanation is that the vHPC activation of PV-INs mediated feedforward inhibition in mPFC is involved in other complex behaviors, such as social memory (Phillips et al., 2019; Sun et al., 2020), as described below, rather than spatial working memory.

#### Amygdala Inputs Drive Feedforward Inhibition in the mPFC

Basolateral amygdala is a universally acknowledged regulatory center of emotional behaviors rather than participating in cognitive functions. Few studies have explored the role this pathway plays in high-order cognitive functions such as decision-making and goal-directed behaviors (Bechara et al., 1999; Ghods-Sharifi et al., 2009). Previous studies also indicated that the BLA interacts with the mPFC in regulating glucocorticoid effects on working memory impairment (Roozendaal et al., 2004) and memory consolidation (Roozendaal et al., 2009). BLA mainly impacts cognition through its tight control of impulsive behaviors (Yin et al., 2019), whereas the subthalamic nucleus-projection-defined prefrontal pyramidal neurons suppress impulsive behavior (Li et al., 2020). However, whether and how these two brain regions coordinate to influence high-order cognitive functions like working memory and spatial memory and the potential roles prefrontal INs may play are still open questions.

### Social Cognition

The mPFC exerts robust control on cognition not only in working memory, but also in social interaction. Social interaction is a complex behavior that requires the coordination of social learning, social memory, and cognitive skills (Bachevalier and Mishkin, 1986; Courchesne et al., 2004; Yizhar et al., 2011; Brumback et al., 2018). Major psychiatric disorders, including depression, autism, SZ, and social anxiety disorder (SAD), all share impaired sociability as a distinctive feature, bringing hefty economic and affection burden on patients and their families (American psychiatric association, 2013). Social behavior performance is influenced by many social skill domains, including social memory, social recognition, and affective discrimination. The mPFC is a key node of the social neuronal network – the social brain (Bicks et al., 2015; Lieberman et al., 2019). Particularly, GABAergic deficits appear to be a convergent point for understanding the neural mechanism of social dysfunction in neuropsychiatric disorders. Imbalanced E/I ratio has been seen in multiple ASD animal models (Rubenstein and Merzenich, 2003; Chao et al., 2010; Han et al., 2012; Gogolla et al., 2014; Karayannis et al., 2014; Vogt et al., 2015; Inan et al., 2016; Antoine et al., 2019). Both PV- and SST- INs contribute to the progress of these psychiatric disorders. For example, knockdown of either PV or SST in the mPFC impaires social interaction performance by dramatically decreasing the interaction time (Perez et al., 2019). Notwithstanding, these two types of medial ganglionic eminence-derived interneurons distinctly control different components of social behaviors. Early life stress, a common risk factor for numerous psychiatric disorders such as SZ, anxiety, and autism, causes social deficits in a sex-specific manner, with a significant loss of PV-INs in the mPFC in juvenile female but not male mice (Holland et al., 2014). Juvenile social isolation preferentially diminishes the activity of PV-INs in the mPFC during social approach in adulthood (Bicks et al., 2020), indicating that early life disturbance triggers social deficits are dominantly mediated by prefrontal PV-INs. In contrast, the function of SST-INs social behaviors differs based on the identify of the social conspecifics. Oxytocin receptor-positive SST-INs, a subset of SST-INs in the mPFC, specifically regulate sexual social behaviors, and inhibiting this group of INs in female mice reduces their interaction with male mice (Nakajima et al., 2014). However, disinhibition of prefrontal SST-INs through inhibition of vasoactive intestinal peptide INs, which are derived from caudal ganglionic eminence, decreases social preference (Koukouli et al., 2017). *In mice*, knockout of *Pten*, a high-risk autism gene, preferentially reduces the intensity of SST-INs, increasing PV/SST ratio, although not specifically in the mPFC (Vogt et al., 2015). Therefore, both PV- and SST- INs in the mPFC play important, complex roles in regulating social cognition and this section will reveal how MD, vHPC, and BLA afferents uniquely and differentially regulate this function.

#### MD-mPFC Pathway Appears to Be Important for Social Preference and Dominance but Not Social Memory and Social Fear

Reduced functional connectivity between MD and mPFC is found to be a major pathological mechanism underlying cognitive dysfunctions, including social deficits (Block et al., 2006; Foss-Feig et al., 2017). It has been found that both mPFC and MD are activated when rats are performing social interaction behaviors (Jodo et al., 2010). As the mPFC and MD are reciprocally connected, concurrent activation of these two brain regions may occur during a social interaction task. Studies in our lab reported that inhibiting MD led to a reduction in social preference in rats (Ferguson and Gao, 2018a,b). In the three-chamber sociability test, rats subjected to MD inhibition via inhibitory DREADDs spend less time in the social chamber containing a novel rat compared to the control group. This behavioral deficit is successfully rescued by elevating PV-INs activity through a PV-promoter-driven excitatory DREADD (Ferguson and Gao, 2018a). This evidence demonstrates that PV-INs play a critical role in mediating the effects of the MD-mPFC pathway in social behaviors. Therefore, the MD-mPFC pathway is involved in regulating not only working memory via PV-INs, but also social interaction. However, it is not clear whether MD-mPFC pathway effects on SST-INs are also important for social interaction. Although prefrontal SST-INs play a powerful control of social fear (Xu et al., 2019), it is unclear whether activating mPFC SST-INs would similarly rescue the MD inhibition-induced social interaction deficit.

#### vHPC-mPFC Connections Regulate Social Memory via PV-INs

As one of the key brain regions for memory consolidation and social memory, the vHPC is essential for the recall of social memory during social interaction. Studies in both humans (Tavares et al., 2015) and rodents (Hitti and Siegelbaum, 2014; Okuyama et al., 2016; Meira et al., 2018) found that vHPC activity is highly involved in social skills requiring social memory. The vHPC appears to be an essential brain region in keeping the memory of familiar conspecifics through a close connection with multiple upstream inputs and downstream target brain regions (Okuyama et al., 2016). For example, the vHPC receives direct inputs from the dCA2 to maintain social memory (Hitti and Siegelbaum, 2014; Meira et al., 2018). The outputs originated from vHPC innervate nucleus accumbens shell to regulate social discrimination (Okuyama et al., 2016). The mPFC is another downstream target recruited by the vHPC to regulate social memory. Due to the abundant projections the vHPC sends to the mPFC, the social-related memory might be retrieved by vHPC-mPFC to help guide social interaction. The mPFC-projecting vHPC neurons are selectively activated when encounter to a live mouse rather than a toy mouse. By combining retrograde tracer with c-fos immunostaining, a study has found that the c-fos expression is significantly higher in mPFC-projecting vHPC neurons in the social interaction trained group comparing with the control group (Phillips et al., 2019). Chronically enhancing the mPFC-projecting neurons in the vHPC significantly impaires social memory retrieval, suggesting a negative correlation between social memory and the vHPC-mPFC pathway activity. Prefrontal neurons are very diverse in responding to social exploration. There are both ON and OFF ensembles tuned to social performance, which requires selectively activation and inhibition of the local circuit that is controlled by inhibitory neurons, although their specific role in social cognition remain unexplored (Liang et al., 2018). Imbalanced E/I ratio has appeared to be a keynote in understanding the pathological mechanism of social deficits underlying multiple psychiatric disorders (van Heukelum et al., 2019). Disrupting the E/I balance in the mPFC mimics autism social deficits featured with severe social defeats (Yizhar et al., 2011) and social cognition in SZ (Bicks et al., 2015). Long-range vHPC afferents to the mPFC innervate both major types of INs (Sun et al., 2019). However, PV-INs appear to be a more important regulator of functional vHPC-mPFC connection in social performance (Alvarez et al., 2020; Sun et al., 2020). Both PV-IN deficiency and impaired social behaviors were seen in SZ model, in which vHPC-mPFC show impaired functional connectivity (Mukherjee et al., 2019). Selective activation of pyramidal neurons disrupted social preference whereas activation of PV-INs via optogenetic method in the mPFC rescues social preference deficit (Yizhar et al., 2011). Phillips et al. (2019) have identified that mPFC projecting vHPC neurons not only innervate pyramidal neurons but also PV- and SST-INs in the mPFC. These exciting results intrigue the hypothesis that increasing activity of mPFC projecting neurons in the vHPC may disrupt the social memory retrieval through enhancing the feedforward inhibition. Still, the detailed function a specific IN subpopulation plays in gating vHPC inputs in social memory requires further studies in the future.

#### BLA-mPFC Pathway Plays an Important Role in Social Cognition but the Involvement of GABAergic INs in the mPFC Remains to Be Determined

The implication of amygdala in social memory is supported by evidence collected from both primate (Kling and Cornell, 1971; Kling and Steklis, 1976; Machado et al., 2008) and rodent studies (Bunnell et al., 1970; Jonason and Enloe, 1971; Sanders and Shekhar, 1995). Beside to MD and vHPC, the participation of mPFC in social interaction behaviors also subjects to the modulation of the excitatory input from BLA (Felix-Ortiz et al., 2016) although its role in social cognition remains untested. BLA inputs to the mPFC function as a suppressor of social exploration (Felix-Ortiz et al., 2016). Stimulation of BLA induces disynaptic response in prefrontal neurons with shorter latency of the inhibitory current (Dilgen et al., 2013). Therefore, the decrease of social interaction following activation of BLA axon terminals in the mPFC might result from elevated inhibition driven by the BLA. Both SST- and PV- INs mediate the BLA-driven feedforward inhibition. Due to distinct biophysical properties of excitatory synapses on these two types of INs, BLA synaptic transmission is facilitated in SST-INs but depressed in PV-INs. These could further create two temporal windows for BLA-mediated inhibition in the mPFC (McGarry and Carter, 2016). Therefore, these two parallel inhibitory pathways might contribute to different influences on social behaviors but further study in the context of social memory is warranted.

### Fear Conditioning Expression and Extinction

Fear is an unpleasant emotion when the subject is aware of the presence of danger, and functions to keep animals alert to avoid harm. However, fear can be maladaptive when normal stimuli are detected as dangerous. The mPFC serves as a top-down regulating center of emotional behaviors, including conditioned fear expression. In the past few decades, plenty of research was devoted to better understand the role of mPFC in the process of conditioned fear acquisition, expression, and extinction, as recently reviewed (Giustino and Maren, 2015; Gourley and Taylor, 2016). Temporal inhibition or permanent lesion of the mPFC significantly reduces the freezing behaviors in the fear-conditioned group in rats (Frysztak and Neafsey, 1991, 1994; Resstel et al., 2006). In contrast, elevating the neural activity of mPFC enhances fear expression and memory formation following the conditioning stimulus (CS) (Shibano et al., 2020). Etkin et al. (2011) elaborate on the recruitment of ACC and mPFC, two subdivisions of the PFC in emotion regulation and found that the activation of mPFC was seen in the fear expression, retention, and extinction, suggesting that mPFC conveys both safety and danger information. More specifically, as reviewed by Giustino and Maren (2015), the prelimbic (PL) and infralimbic (IL) subdivisions of the rodent mPFC respectively regulate the expression and suppression of fear in rodents. Under certain conditions, the PL and IL act in concert, exhibiting similar patterns of neural activity in response to aversive conditioned stimuli and during the expression or inhibition of conditioned fear; albeit these mPFC subdivisions may code opposing behavioral outcomes, with PL biased toward fear expression and IL toward suppression (Giustino and Maren, 2015). The question raised by this intriguing hypothesis is whether and how this opposing action is achieved.

Recently, the functional importance of mPFC GABAergic INs in regulating conditioned fear expression has been evidenced. Calcium activity of prefrontal SST-INs in mice is increased during and after the conditioning, suggesting that the activity of SST-INs in the mPFC may underlie the fear acquisition and memory consolidation (Cummings and Clem, 2020). Furthermore, photoactivation of SST-INs increases freezing behaviors during memory retrieval. Conditioning pair electrical shock with neutral stimulus enhances the inhibition from SST-INs to PV-INs, resulting in the disinhibition of PNs in the mPFC (Cummings and Clem, 2020; Flores-Barrera et al., 2020). MK-801 (an NMDAR antagonist) treatment primarily diminishes the GABAergic transmission and increases excitation and inhibition ratio (Flores-Barrera et al., 2020). However, the dysfunction of inhibitory transmission does not disrupt the acquisition of fear conditioning (Flores-Barrera et al., 2020). Instead, it enhances fear memory retrieval and impairs extinction. Direct infusion of GABAa receptor agonist generates an opposite effect on freezing response (Flores-Barrera et al., 2020). These findings depict a local disinhibition circuit onto excitatory pyramidal neurons through SST-mediated dendritic inhibition and PV-mediated perisomatic inhibition, respectively. In the following sections, we will review and summarize how primary long-range afferents regulate this disinhibition circuit in the mPFC in fear conditioning.

#### MD-mPFC Pathway for Both Fear Expression and Extinction

Both MD and mPFC are main structures in controlling fear expression and extinction (Oyoshi et al., 1996; Herry and Garcia, 2002; Li et al., 2004; Padilla-Coreano et al., 2012). In the establishment of conditioned fear, MD serves as a relay to transfer information from the superior colliculus to both the mPFC and amygdala. Tonic but not burst activity of MD is required for the fear extinction induced by alternative bilateral visual stimulation, a task known to promote fear extinction by increasing visual attention (Baek et al., 2019). Enhancing burst firing by knocking out phospholipase C-beta4 abolishes the effect of alternating bilateral stimulation-induced attenuation on fear relapse (Baek et al., 2019). In contrast, the activity of BLA neurons is inhibited by the alternating bilateral stimulation. MD drives feedforward inhibition in the BLA to support long-lasting fear attenuation (Baek et al., 2019). Surprisingly, the role of MD-mediated regulation of GABAergic INs in the mPFC and other fear-associated brain regions has yet to be examined for fear expression and extinction behavior. It has been shown that activating MD afferents in the BLA induces monosynaptic EPSC and disynaptic IPSC (McGarry and Carter, 2017), but the specific IN subtypes in the BLA gating MD inputs, and more relevant to the MD-mPFC inputs, during a different fear conditioning phase, remains to be explored.

#### vHPC-mPFC in Social Fear

Social fear is one type of maladaptive fear shown in SAD. Patients usually experience intense social anxiety, which severely affects their daily life. In rodents, social fear can be induced by a social defeat paradigm through the resident-intruder test, followed by social interaction test to measure the fear memory (Qi et al., 2018). Both mPFC and vHPC are involved in the regulation of social behavior. However, controversy about this pathway’s complex function in the expression of social fear has remained due to several reasons. First, the vHPC inputs innervate both excitatory and inhibitory neurons in the mPFC. This broadens the range of diversity of the role the vHPC-mPFC pathway plays in controlling social fear. Second, the reported activity of the mPFC in social fear expression is controversial. For example, after social defeat, the expression of early response genes suggests that the suppression of mPFC activity can last for 7 days from the initial social fear induction (Qi et al., 2018), emphasizing the inhibition of mPFC is essential for fear memory maintenance. However, *in vivo* recording of prefrontal neuron activity discovers an increase of firing rate of pyramidal neurons in the PL region of mPFC during social fear expression (Abe et al., 2019; Xu et al., 2019). Interestingly, activating SST-INs, which are known to inhibit PV-INs, facilitates the expression of social fear. SST-INs in the mPFC participate in conditioned social fear behavior by disinhibiting excitatory networks via suppressing PV-INs activity (Xu et al., 2019). Indeed, SST-INs have been reported to gate the vHPC input in the mPFC. PFC projecting vHPC neurons show higher activity during a social encounter (Phillips et al., 2019). Therefore, during the resident-intruder test, the vHPC activity may drive SST-INs to disinhibit the prefrontal network activity via PV-INs. However, during social encounters following the social fear conditioning, the BLA may inhibit both vHPC and mPFC to allow stress expression and enhance social fear memory (Qi et al., 2018). Further experiments are needed to explore the specific roles of prefrontal INs in regulating vHPC-mPFC pathway-dependent social interaction.

#### vHPC-mPFC Connection Drives Fear Extinction via PV-INs

Preventing the renewal of fear following extinction could be a novel and effective clinical intervention procedure for treating maladaptive learned fear responses. The expression of a conditioned stimulus (CS)-evoked fear during the extinction is a context-dependent fear renewal, which involves re-expression of fear when encountering the CS outside the extinction context. Previous studies reported that context-independent expression of extinct fear could be induced by inactivating the vHPC (Corcoran and Maren, 2001; Hobin et al., 2006; Zelikowsky et al., 2013), suggesting that vHPC might be a fear suppressor. A study conducted by Marek et al. (2018) supports this notion. The researchers find that pharmacologically activating the vHPC input to the mPFC results in a decrease of freezing behavior in the extinction context (Marek et al., 2018). More importantly, they discover that the vHPC controls fear relapse through feedforward inhibition of amygdala-projecting prefrontal pyramidal neurons (Marek et al., 2018). The IL region of the mPFC is critical for fear relapse. Activating IL axon terminals in the amygdala inhibits the re-expression of fear when encountering the CS. Interestingly, a subsequent *ex vivo* study discovers that vHPC projections primarily target inhibitory neurons in L2/3 of the IL. Moreover, these INs mainly comprise PV- rather than SST-INs, as inhibiting SST- INs in the IL does not affect vHPC evoked feedforward inhibition on IL pyramidal neurons (Marek et al., 2018). Together, although both SST- and PV- INs in the IL receive vHPC inputs, PV-INs are more important than SST- INs in regulating the vHPC-mPFC pathway in fear relapse.

#### BLA-mPFC in Fear Acquisition, Expression, Extinction

Although BLA is the hub for emotional learning, the mPFC serves as a suppressor to control maladaptive fear learning (Likhtik and Paz, 2015). The communication between the amygdala and the mPFC is critical for expressing learned fear (Likhtik et al., 2013; Likhtik and Paz, 2015). The synaptic transmission from BLA to mPFC can be blocked by selectively stimulating the BLA inputs at high frequency, and blocking the BLA-mPFC pathway activity attenuated the conditioning-stimulus evoked increase of firing in mPFC (Klavir et al., 2017). This effect suggests that BLA projection transmits learned CS-US associations to the mPFC (Garcia et al., 1999; Sotres-Bayon et al., 2012; Senn et al., 2014). The function of prefrontal INs in regulating the amygdala-mPFC pathway in this behavioral performance remains to be fully characterized. Given the diversity of IN subpopulations in the mPFC, each phase of the fear conditioning process might be differentially regulated by GABAergic INs in a cell-type-specific manner. Indeed, a recent study reported that the presentation of CS inhibited PV-INs to disinhibit pyramidal neurons in the mPFC to drive fear expression (Courtin et al., 2013). Theta oscillation, which is coupled with the presentation of CS (Likhtik et al., 2014), is enhanced by the inhibition of PV-INs (Courtin et al., 2013). The inhibition of PV-INs may originate from the activation of SST-INs, which inhibit both excitatory and inhibitory neurons in the mPFC. Indeed, CS paired with footshock enhances not only the activity of SST-INs but also the inhibition of PV-INs in the PL (Cummings and Clem, 2020). Interestingly, the synaptic transmission BLA formed on both SST-INs and pyramidal neurons are enhanced, while the connection between BLA inputs to PV-INs is weakened. This finding fills the blank of how BLA inputs regulate the fear conditioning association through mPFC inhibitory circuits mediated by both SST- and PV-INs.

**FIGURE 2:**
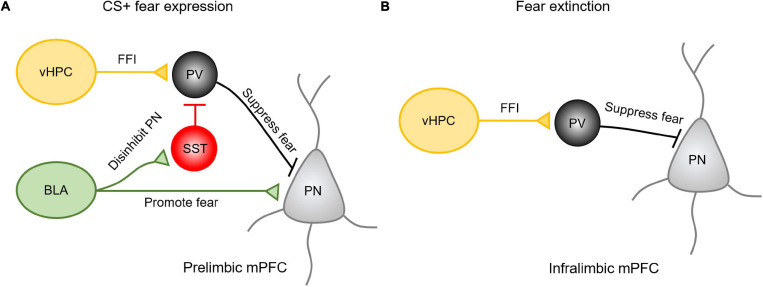
The vHPC and amygdala inputs in the mPFC exhibit regional specificity and differential mechanisms in regulating fear expression and extinction. **(A)** Upon presenting conditioned cues, BLA inputs could activate projecting pyramidal neurons in the PL to drive fear expression or activate SST-INs to disinhibit pyramidal neurons in promoting the fear expression. Meanwhile, vHPC inputs drive feedforward inhibition (FFI) by recruiting PV-INs to prevent these projecting neurons from the fear response. **(B)** During extinction, vHPC inputs drive feedforward inhibition onto the amygdala projecting neurons in the IL to suppress the relapse of fear.

How are vHPC and amygdala inputs integrated into the PL and IL subregions in the mPFC to influence fear expression and extinction? The existing evidence shows that in responding to the presence of conditioned cues, BLA inputs primarily activate projecting pyramidal neurons in the PL to drive fear expression (Sotres-Bayon et al., 2012) (Figure 2A). The mechanism for this effect may be explained by the fact that after learning a CS-US pairing, BLA afferent activity favors the recruitment of SST-INs over PV-INs, resulting in the disinhibition of pyramidal neurons and the promotion of fear expression (Cummings and Clem, 2020). At the same time, vHPC afferents drive feedforward inhibition by recruiting PV-INs to prevent the activation of projecting neurons from the fear response. Therefore, vHPC inputs are believed to gate the BLA inputs via prelimbic PV-INs (Sotres-Bayon et al., 2012). In contrast, the extinction of fear is predominantly regulated by vHPC-driving feedforward inhibition onto the amygdala projecting neurons in the IL (Sotres-Bayon et al., 2012; Marek et al., 2018). Activation of vHPC increases the activity of PV-INs in the IL to suppress the prefrontal-amygdala projecting neurons (Figure 2B). These studies suggests that multiple long-range afferents may cooperate to regulate distinct complex behavior by activating specific cell types in a region-specific manner.

However, it is important to note that when it comes to fear conditioning, different modes of fear acquisition recruit different components of mPFC microcircuitry. Evidence shows that levels of freezing response are sensitive to the expression of PV in the mPFC (Caballero et al., 2020) or local disruption of vHIP and BLA inputs (Miguelez Fernández et al., 2021). For example, the amygdalar inputs in the mPFC are regulated by the vHPC inputs-driving feedforward inhibition (Caballero et al., 2014). The recruitment of vHPC and amygdalar inputs in fear expression and/or extinction is also regulated by PV-IN activity magnitude (Caballero et al., 2020). Specifically, although vHPC recruitment of PV interneurons can suppress fear expression in contextual fear conditioning, reducing PV expression by 25% in the mPFC through shRNA knockdown in adolescence has no effect on the expression of conditioned fear when using a trace-fear conditioning paradigm (Caballero et al., 2020). Prefrontal infusion of a7nAChR antagonist methyllycaconitine also differentially modulates the gain of vHPC and amygdalar inputs and fear responses in an age-dependent manner (Miguelez Fernández et al., 2021).

## Conclusion and Future Directions

In this review, as summarized in Figure 1, we dissect the anatomical connection the MD, vHPC, and BLA formed with PV- or SST- INs in the PFC. All three brain regions form feedforward inhibition in the mPFC by targeting certain IN subpopulations. The MD mainly innervates PV-INs. Activation of the MD-PFC feedforward inhibitory circuit is critical for working memory performance and social preference. However, although SST-INs in the mPFC participate in the encoding of working memory, social interaction, and fear expression, what roles they play in the mPFC in gating MD inputs in these complex behaviors remains ambiguous. It is still unclear whether MD inputs form functional synaptic connections with SST-INs in the mPFC.

In contrast, it has been shown that the vHPC sends projection onto both PV- and SST- INs in the mPFC. These two subpopulations of INs separately regulate vHPC-mPFC control of distinct complex behaviors. SST-INs facilitate the coherence between prefrontal single-unit activity with vHPC theta oscillation during the sample phase of the spatial working memory task. Afferents from the vHPC target PV-INs in the IL to inhibit CS-induced fear renewal, and these connections also appear to be important for social cognition. When it comes to social fear expression, vHPC regulation of SST-INs may play a more important role.

The BLA-mPFC pathway plays an essential role in social cognition and fear acquisition, expression and extinction, but the specific mechanisms remain unclear. Although BLA inputs innervate both PV- and SST- INs to drive feedforward inhibition to the local prefrontal circuit, the unique functions of these feedforward inhibition driven by the BLA-mPFC pathway is largely understudied. The BLA inputs in the mPFC mainly serve as a suppressor of social exploration, which may require the feedforward inhibition driven by BLA inputs. In the expression of conditioned fear, synaptic transmission the BLA formed onto SST-INs is enhanced, which could inhibit PV-INs and disinhibit pyramidal neurons in the mPFC.

Dissecting the function of these long-range inputs that drive feedforward inhibition in a cell-type-specific manner is valuable for understanding the mechanisms underlying multiple behavioral impairments associated with neuropsychiatric diseases and potential novel therapeutic targets for better control over pathological development. However, outstanding questions are raised to be addressed in future studies.

• Although functional overlaps exist among different PFC-associated pathways, each IN subtype may be preferentially coupled with specific components of complex behaviors when engaged by distinct afferents. However, cell type-specific regulation of PFC-associated behaviors is overly understudied. For example, the function of BLA-prefrontal GABAergic pathways in cognitive behaviors other than emotional control is barely explored.
• One of the prominent shared features of PFC-associated psychiatric disorders is the developmental onset of pathology. However, the time course of each type of psychiatric disorder, such as SZ and ASD, are not identical. The onset of SZ could vary between 12 and 14 years old and encounter the first peak from 15 to 30 years old, which is adolescence and young adult age (Häfner, 2019). In contrast, ASD could be presented in the first 18 months of life (Zwaigenbaum et al., 2021), which may be phase-locked with the developmental trajectories of specific interneuron populations. For example, disturbance of interneuron development during adolescence may have a stronger effect on the onset of SZ. Furthermore, embryonic and early life development, including the processes of proliferation and migration, may underlie the pathology of ASD. Therefore, characterizing the developing features of each subtype of INs, and the long-range pathway they dominantly regulate is essential for a better understanding of the pathological process.
• GABAergic INs, even within the same subtype, are highly heterogeneous based on physiological and biological features. PV-INs can be divided into two subtypes according to their morphology-basket cells and chandelier cells. SST-INs also consist of few subtypes which possess distinct physiological properties - Martinotti cells, and non-Martinotti cells. The functional differences among these small subgroups are not well studied yet. For example, in the phenomenon of cell loss seen in ASD, the population of basket cells remain relatively stable, while chandelier cells subject to a persistent decrease of intensity (Ariza et al., 2018). It thus increases the difficulty to fully understand the properties of each long-range pathway connected with the mPFC and their functions in driving complex behaviors. Bottom-Up research combining *in vivo* active cell labeling markers with molecular and electrophysiological techniques may be helpful to address this question.
• Finally, the mechanisms underlying different long-range afferents’ abilities to activate distinct IN subpopulations for distinct behaviors remain unknown. For example, vHPC innervates SST- and PV- INs, but vHPC control of PV-INs is important for fear renewal or social interaction. In contrast, vHPC control of SST-INs is vital for spatial memory. Understanding how a particular input is able to engage distinct subpopulations of INs to regulate different behaviors is a great unknown question for future study.

## Author Contributions

S-SY, NM, YS, and W-JG wrote and edited the manuscript. All authors contributed to the article and approved the submitted version.

## Conflict of Interest

The authors declare that the research was conducted in the absence of any commercial or financial relationships that could be construed as a potential conflict of interest.

## Abbreviations

ACC: anterior cingulate cortex
BLA: basolateral amygdala
DNMST: delayed non-match to sample T-maze task
DREADDs: designer receptors exclusively activated by designer drugs
EPSCs: excitatory postsynaptic currents
FFI: feedforward inhibition
GABA: gamma-aminobutyric acid
IL: infralimbic
INs: interneurons
L1: layer 1
MD: mediodorsal thalamus
mPFC: medial prefrontal cortex
PL: prelimbic
PTSD: post-traumatic stress disorder
PV: parvalbumin
SAD: social anxiety disorder
SST: somatostatin
SZ: schizophrenia.
